# Extracellular vesicles: messengers of cross-talk between gastric cancer cells and the tumor microenvironment

**DOI:** 10.3389/fcell.2025.1561856

**Published:** 2025-04-16

**Authors:** Xiwen Li, Xian Lu, Mi Liu, Junjie Chen, Xirong Lu

**Affiliations:** ^1^ Kunshan Hospital of Chinese Medicine, Affiliated Hospital of Yangzhou University, Kunshan, China; ^2^ College of Pharmaceutical Sciences, Soochow University, Suzhou, Jiangsu, China; ^3^ Jiangsu Province Engineering Research Center of Precision Diagnostics and Therapeutics Development, Soochow University, Suzhou, China; ^4^ Department of Clinical Medical Research Center, Affiliated Hospital of Nantong University, Nantong, China

**Keywords:** gastric cancer, exosome, tumor microenvironment, regulation, interaction

## Abstract

Gastric cancer is a common malignancy characterized by an insidious onset and high mortality rate. Exosomes, a special type of extracellular vesicle, contain various bioactive molecules and have been found to play crucial roles in maintaining normal physiological functions and homeostasis in the body. Recent research has shown that the contents of exosome play a significant role in the progression and metastasis of gastric cancer through communication and regulatory functions. These mechanisms involve promoting gastric cancer cell proliferation and drug resistance. Additionally, other cells in the gastric cancer microenvironment can regulate the progression of gastric cancer through exosomes. These include exosomes derived from fibroblasts and immune cells, which modulate gastric cancer cells. Therefore, in this review, we provide a brief overview of recent advances in the contents and occurrence mechanisms of exosome. This review specifically focused on the regulatory mechanisms of exosomes derived from gastric cancer and other cellular subtypes in the tumor microenvironment. Subsequently, we summarize the latest research progress on the use of exosomes in liquid biopsy, discussing the potential of gastric cancer exosomes in clinical applications.

## 1 Introduction

Gastric cancer (GC) was ranked as one of the most common cancers globally. Due to its insidious onset, diagnosis often occurs at an advanced stage, contributing to a high mortality rate (Smyth et al., 2020). Studies indicated that more than one million people are diagnosed with gastric cancer each year, with a higher incidence in East Asia, possibly linked to dietary habits and lifestyle factors (Oliveira et al., 2015; Lee et al., 2016; Thrift and El-Serag, 2020). Therefore, early detection of treatable GC is critical to improving patients’ long-term prognosis, underscoring the need for the development of innovative noninvasive biomarkers with high specificity and sensitivity for early GC screening.

Various classification systems exist for GC. As early as 1971, Japanese scholars classified early GC into three types based on morphological features: protruding, superficial, and depressed. The Paris endoscopic classification supplemented this, incorporating contributions from research groups worldwide (Participants in the Paris Workshop, 2003). Other classification methods include the Bormann classification and the Lauren classification (Yang et al., 2007; Panani, 2008). Researchers commonly use histological classification when studying the pathophysiological mechanisms of GC, with 90% of cases exhibiting adenocarcinoma. In clinical practice, the TNM staging system is employed to assess the progression of GC and guide appropriate treatment. Although new staging methods have been proposed after neoadjuvant therapy, further clinical validation is needed. Molecular advances have increased our understanding of GC biomarkers (Chia and Tan, 2016), and single-cell techniques may offer insights into molecular and biological markers for future classification and staging, although these methods have not yet been standardized. Advancements in medical technology, increased health awareness, and the development of diagnostic tools such as endoscopy, novel targeted drugs and immunotherapy have contributed to a gradual decline in the diagnosis and mortality rates of GC. The increasing average lifespan and progress in healthcare suggest that cancer, including GC, may become more prevalent as a chronic disease in the future (Sarfati et al., 2016; De Magalhaes, 2013). Early diagnosis, reducing mortality, and improving patients’ quality of life pose key challenges for future healthcare professionals and researchers. This underscores the importance of in-depth research into GC, especially exploring its pathogenesis and developing new diagnostic methods to address future challenges (Rocken, 2023; Zhang C. et al., 2023; Sugano et al., 2023).

**FIGURE 1:**
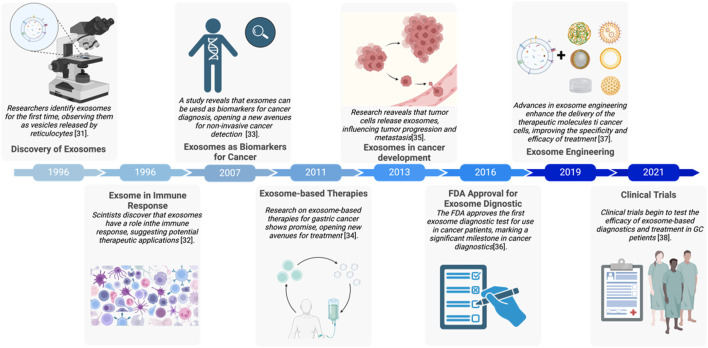
Major events of exosomes in gastric cancer development (Raposo et al., 1996; Chaput et al., 2005; Kok and Yu, 2020; Zhang Y. et al., 2020; Azmi et al., 2013; Masyuk et al., 2013; Liang et al., 2021; Rezaie et al., 2022). The figure was created by Biorender (https://www.biorender.com/).

Exosomes are a class of Extracellular Vesicles (EVs) produced by donor cells, which can be used as an effective intercellular communication tool to transmit a series of molecules to recipient cells (Kalluri and LeBleu, 2020; Zhang M. et al., 2023)**.** Exosomes are normally around 30 nm–150 nm. On the surface of exosomes, proteins or recognition components that enhance their targeting ability can potentially aid in exosomes evasion of recognition and attack by the immune system (Yang et al., 2019). Exosomes were initially considered platelet dust, vesicles involved in the cellular excretion of metabolic waste (Couch et al., 2021). However, current research indicates that exosomes possess the following characteristics: they play a highly targeted role in cellular communication and participate in physiological activities of cells, including the regulation of cell growth and apoptosis (Wu et al., 2022). In studies related to exosomes and cancer, exosomes were shown to influence the metastasis and growth of tumors, including the regulation of immune cell functions (Liu et al., 2016; Li M. Y. et al., 2021). Additionally, tumor cells can transfer certain characteristics, such as drug resistance, through exosomes (Mashouri et al., 2019) and the identities of the tumor cells could be reflected in the exosomal cargos. As substances secreted by almost all cells, exosomes from different cell sources exhibit functional diversity; for instance, immune cells can regulate the growth of tumor cells through exosomes (Xu et al., 2020). Currently, exosomes play a crucial role in the occurrence and development of several cancers, such as lung cancer, pancreatic cancer, and liver cancer (Ariston Gabriel et al., 2020; Wang et al., 2019; Khan et al., 2023). Similarly, exosomes also impact on the occurrence and development of GC. Furthermore, exosomes can be maintained steadily in a variety of body fluids because the lipid bilayer structure adequately shields the contents from degradation, which suggests that exosomes could be an effective option for dependable biomarkers (Wu et al., 2021). Exosomes and their derived cargos have been exploited as new indicators for cancer evaluation and prediction, as evidenced by an increasing volume of examples in the literature (Tang et al., 2021). Exosomes have also been utilized for cancer treatment to transport biological materials and chemotherapeutic medications, due to its transport and stability characteristics. For instance, Exosomes are thought to be effective delivery systems for RNA-based treatment approaches, which open a new horizon for nano-therapeutic strategies in caner field (Zhang M. et al., 2023; Wandrey et al., 2023). Exosomes that have been edited to include internal therapeutic compounds and surface ornamentation are commonly referred to as engineered exosomes. Engineered exosomes may effectively and accurately carry anticancer medications to tumor locations with fewer treatment-related side effects (Figure 1) (Sadeghi et al., 2023; Zhang F. et al., 2023).

This article primarily summarizes recent advances in exosome-related research in the field of basic GC, including the regulation of tumor growth by tumor-derived exosomes, the interplay between tumor microenvironment (TME) and tumor-derived exosomes, and the study of GC exosomes in clinical applications. Finally, we discuss engineered strategies for exosome therapy and provide an outlook on future research findings.

## 2 Classification and main contents of exosomes

### 2.1 Difference between exosomes and microvesicles

In fact, exosomes are a subtype of EVs produced by donor cells exosomes. The EVs mainly include exosomes and microvesicles, and if we do not consider their biological functions, their differences are primarily reflected in their origin and size. Microvesicles are formed by budding directly from the plasma membrane; these vesicles have diameters ranging from 50 to 500 nm and can even reach 1 μm. Exosomes, on the other hand, are formed by the fusion of multivesicular bodies (MVBs) with the plasma membrane, releasing intraluminal vesicles (ILVs) from the luminal cavity of MVBs, resulting in exosomes with diameters ranging from 30 to 150 nm (van Niel et al., 2018). More than two fold as many exosomes are found in the blood of cancer patients than in the blood of healthy people, highlighting the importance of exosomes and their very active intercellular communication in cancer (Zhang and Yu, 2019). Some cell types have been found to employ unique methods for exosome generation; for example, T cells can produce exosomes on the cell surface by utilizing the plasma membrane (Booth et al., 2006). It is noticeable that microvesicles and exosomes are overlapping in size and have similar appearances, so exosomes are primarily identified by marker proteins.

### 2.2 Exosomes play different functions depending on source and cargoes

Exosomes from different sources exhibit distinct biological functions, which may be related to the specific structures of the source cells, and they also differ in their targeting ability (Zhang M. et al., 2023; Gao et al., 2018). Furthermore, in practical research, the process of extracting exosomes may involve other vesicle structures, such as apoptotic bodies and ectosomes, collectively leading to the formation of extracellular vesicles (Willms et al., 2016).

**FIGURE 2:**
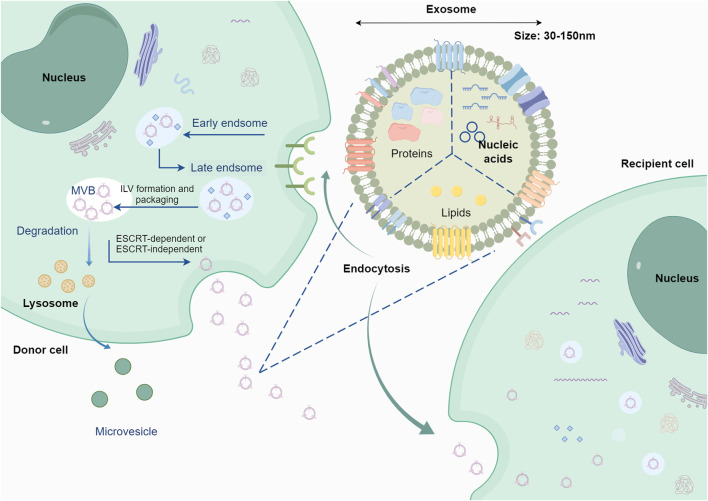
Origin and cargo of exosomes. Following the processes of early endosomes and late endosomes, exosomes are formed through the fusion of multivesicular endosomes with the plasma membrane, releasing intraluminal vesicles into the lumen of the multivesicular endosome. Exosomes contain various cargoes associated with the parent cell, including nucleic acids, proteins, lipids, etc. The figure was created by Figdraw (www.figdraw.com).

Exosomes biogenesis initiates with endosome formation, which occurs when cells internalize extraneous materials via endocytosis. Over time, early endosomes progress into late endosomes, giving rise to endocytic vesicles through membrane invagination. These vesicles become enclosed within endosomes and ultimately develop into MVBs, a pivotal stage in exosomes generation. Endocytic vesicles transport distinct proteins, RNA, lipids, and various molecules (Kalluri and LeBleu, 2020). Exosomes biogenesis initiates with endosome formation, which occurs when cells internalize extraneous materials via endocytosis. Over time, early endosomes progress into late endosomes, giving rise to endocytic vesicles through membrane invagination. These vesicles become enclosed within endosomes and ultimately develop into MVBs, a pivotal stage in exosomes generation. Endocytic vesicles transport distinct proteins, RNA, lipids, and various molecules (Colombo et al., 2014). The ESCRT system comprises four main complexes (ESCRT-0, ESCRT-I, ESCRT-II, and ESCRT-III) along with associated cofactors. Responsible for cargo sorting and MVBs formation, ESCRT plays a crucial role. Rab GTPases, such as Rab 27a, Rab 27b, and Rab35 (Ostrowski et al., 2010), govern the transport and fusion of MVBs with plasma membranes. Exosomal cargoes (e.g., miRNAs, proteins, lipids) undergo selective, rather than random, loading through specific mechanisms. miRNAs are regulated by RNA-binding proteins (e.g., hnRNPA2B1, YBX1) or specific sequence signals (Villarroya-Beltri et al., 2013; Garcia-Martin et al., 2022). Proteins are packaged into exosomes through ubiquitination, ESCRT-dependent or ESCRT-independent pathways (Figure 2) (Thery et al., 2001; Li X. et al., 2020). Lipids are sorted via lipid rafts or specific lipid transporters (Crivelli et al., 2022; Wei et al., 2021). Furthermore, heat shock proteins (e.g., HSP70) play a role in endosomal sorting, highlighting the diverse mechanisms involved in their biogenesis (Gobbo et al., 2016).The type and abundance of exosomal contents are typically determined by the state of the donor cells, including the physiological and pathological conditions of these cells. Various interventions may also influence the production of exosomes (Debbi et al., 2022; Jafari et al., 2020). The contents of exosomes include RNA, proteins, lipids, DNA, etc. (Figure 2), and exosomes carrying specific cargo can perform particular functions (Kimiz-Gebologlu and Oncel, 2022; Tenchov et al., 2022). This topic is a primary focus of current exosomes research. Understanding the contents of exosomes is crucial for studying GC, as it helps in comprehending how exosome secretion may vary with changes in TME.

## 3 Interactions of exosomes in the progression of GC

Tumors constantly interact with their surrounding environment through interactions involving immune regulation, such as immune cells attacking tumors. As tumors undergo continuous changes, they could evade immune cell attack (Demaria et al., 2019; Miller and Sadelain, 2015). The interactions between exosomes and tumors are similar to this dynamic. Tumor cells themselves can secrete exosomes, known as tumor-derived exosomes, and surrounding immune cells can also release exosomes. Exosomes from different sources exhibit heterogeneity in their contents and biological functions. For example, tumor-derived exosomes can transfer drug resistance between tumor cells, regulate tumor immune evasion and promote tumor cell growth or metastasis by activating or inhibiting certain signaling pathways of recipient cells through transferring certain chemical compounds to modulate the development of the tumor process (Isaac et al., 2021). Noncoding RNA (ncRNA) is one of the groups of bioactive molecules included in exosomes that work as the main carrier of information transfer between tumors cells. Examples of ncRNA include microRNAs (miRNAs), circular RNAs (circRNAs), and long noncoding RNAs (lncRNAs) (Lakshmi et al., 2021; Chang and Wang, 2019). On the other hand, exosomes from other cell sources, such as immune cell-derived exosomes generated from NK cells and T cells, play a role in killing tumor cells and inhibiting tumor growth (Kang et al., 2021; Zhu et al., 2017). Therefore, when discussing the regulatory role of exosomes in the occurrence and development of GC, it is essential to clarify the source of exosomes and discuss them separately based on their origins. From the perspective of tumor interactions, tumor-derived exosomes may be potential biomarkers for predicting tumor development. From the perspective of tumor killing, immune cell-derived exosomes could be used as therapeutic targets for controlling tumor development.

### 3.1 Research on the key components in GC exosomes that promote cancer proliferation and metastasis

The first study on GC exosomes and their association with tumor proliferation was published in 2009. Qu et al. reported that exosomes produced by the GC cell line SGC7901 could promote tumor cell proliferation by activating the PI3K/AKT and MAPK/ERK pathways (Qu et al., 2009). In comparison to early studies on GC and exosomes, current studies delve deeper into the specific components that play a role in exosomes. In a study by Qiu et al., exosomes produced by GC cells were shown to impact tumor metastasis. They observed significantly elevated levels of miR-519a-3p in the serum of patients with GC and liver metastasis compared to patients without liver metastasis. Researchers have also noted that exosomes produced by GC cells are internalized by macrophages in the liver. MiR-519a-3p in exosomes targeted DUSP2, activating the MAPK/ERK pathway and inducing M2 polarization in macrophages. This process promotes angiogenesis and accelerates GC liver metastasis (Qiu et al., 2022).

Exosomes can deliver miRNAs, and abundant research has been conducted in this area (Sun et al., 2018a). GC exosomes have been found to induce peritoneal fibrosis and mesothelial-to-mesenchymal transition (MMT), promoting GC metastasis (Deng et al., 2017). Researchers identified miRNA-21-5p in exosomes produced by GC cells, which can be internalized by peritoneal mesothelial cells (PMCs). This internalization activates the TGF-/Smad pathway, promoting peritoneal metastasis in cancer (Li et al., 2018). miRNA-106aβ in GC exosomes is believed to promote peritoneal metastasis by regulating Smad7, and the histological basis for this promoting mechanism is disruption of the mesothelial barrier (Zhu et al., 2020; Zhu et al., 2022). miRNA-15b-3p was detected in the serum of GC patients and in exosomes derived from GC cells. It is associated with poor overall survival in patients and enhances GC migration and proliferation by inhibiting the expression of the apoptosis-related proteins caspase-3, caspase-9, and DYNLT1 (Zhu et al., 2022). GC exosomes can also increase adhesion between GC cells and mesothelial cells, promoting tumor metastasis (Arita et al., 2016). These findings suggested that researchers may be able to inhibit GC metastasis by downregulating the expression of specific miRNAs in exosomes.

In comparison to miRNAs, circRNAs exhibit better stability and can function as regulators of gene expression by serving as miRNA sponges (Chen, 2020; Memczak et al., 2013; Hansen et al., 2013). CircRNAs have also been found in exosomes and play crucial roles in cell proliferation and disease progression (Dai et al., 2020; Li C. et al., 2021). Studies have shown that circNRIP1 can be transmitted through GC cell exosomes, where it promotes GC cell proliferation by regulating the AKT1/mTOR pathway. This finding suggested that specific circRNAs carried by GC exosomes can enhance GC cell proliferation (Zhang et al., 2019). Similarly, another study revealed that circITTCH can act as a sponge for miRNA-199a-5p, suppressing GC metastasis by increasing Klotho expression (Wang Y. et al., 2021). CircNEK9 was found to promote GC progression through the miRNA-409-3p/MAP7 axis (Yu et al., 2021). Circ_0004104 was shown to target RNF2, accelerating GC progression (Yue et al., 2021). CircUBE2Q2 was shown to promote malignant progression in GC by mediating autophagy and the glycolytic pathway (Yang J. et al., 2021). In addition, exosome-derived circATP8A1 from GC cells induce macrophages M2 polarization via the circATP8A1/miR-1-3p/STAT6 axis, and tumor progression (Deng et al., 2024). Similarly, exosomal circMAN1A2 competed with FBXW11 for binding to SFPQ, preventing FBXW11-mediated k48-linked ubiquitination and SFPQ protein degradation, thereby stabilizing SFPQ expression.CircMAN1A2 can be encapsulated by hnRNPA2B1 in exosomes and can be taken up by T cells, thus affecting antitumour immunity (Shen et al., 2025). Additionally, circRNAs, including RELL1, regulate g GC progression through the autophagy pathway (Sang et al., 2022).

Exosomes can also carry lncRNAs (Liu R. et al., 2019; Sun et al., 2018b). HOX transcript antisense RNA (HOTAIR) has been detected in the peripheral blood serum and tumor tissues of GC patients, and its overexpression can increase tumor growth and metastatic capabilities (Chen P. et al., 2023; Zhang J. et al., 2020). Similarly, the lncRNA TTN-AS1 promotes GC cell growth and migration by enhancing CDX2 expression (Wang et al., 2023). The lncRNA SPRY4-IT1 regulates cell proliferation and migration in GC by modulating the AMPK pathway (Fan et al., 2019). Exosomes secreted by GC cells containing a lncRNA (lncAKR1C2) can enhance lymph node metastasis in GC (Zhu et al., 2023). Exosomal LINC01480 can promote the proliferation, migration and invasion of GC cells by upregulating VCAM1 expression through competitive binding with miR-204-5p (Zhang Y. et al., 2024). Similarly, exosomal LINC00355 promotes the malignant progression of GC through histone deacetylase HDAC3-mediated TP53INP1 transcriptional inhibition (Zhao et al., 2023).

Proteins in exosomes include membrane proteins and glycoproteins anchored on the surface, and these proteins have been found to play important roles in biological processes (Wang X. et al., 2022; Salunkhe et al., 2020). Exosomes secreted by GC cells contain CD97 on the surface. *In vitro* experiments have shown that knocking down CD97 expression can inhibit tumor metastasis. This promoting mechanism occurs by increasing the expression of epithelial adhesion molecules, and CD97-enriched exosomes can facilitate lymphatic metastasis of GC cells (Shen X. et al., 2022). CD44 in exosomes derived from GC cells has also been found to promote lymphatic metastasis (Wang M. et al., 2022). Exosomes secreted by GC cells can carry epidermal growth factor receptor (EGFR) and integrate EGFR into the plasma membrane of liver cells. This translocation of EGFR from cancer cells to liver cells can inhibit the action of miRNA-26a/b, activate hepatocyte growth factor (HGF), and promote liver metastasis of cancer cells (Zhang et al., 2017). Another example of such a protein is FZD10, which can be identified as a protein in exosomes from various cancers that can sustain the proliferation of GC cells (Scavo et al., 2019). Exosomal proteins are expressed mainly in parent cells, reflecting their biological characteristics. Moreover, membrane proteins such as CD97 can enhance the adhesion efficiency of exosomes, suggesting that regulating the expression of specific proteins on the surface of exosomes can also modulate their function. Therefore, Inhibiting the biological effects of some exosomal proteins can effectively inhibit their growth-promoting and migration-promoting effects on recipient cells.

### 3.2 The regulatory role of GC-derived exosomes in malignant tumor behavior

During the development and treatment of GC, tumor cells continuously adapt through self-renewal or progressive mutation, enhancing their ability to spread and develop resistance to therapeutic drugs. Malignant behaviors such as angiogenesis, epithelial–mesenchymal transition (EMT), and drug resistance in tumor cells are closely associated with the difficulty of cancer treatment and the metastatic potential of tumor cells. Recent research has also revealed the regulatory role of GC exosomes in these behaviors (Yan et al., 2017; Eguchi et al., 2022).

Tumor angiogenesis is a common malignant event in the growth and metastasis of tumors and provides oxygen and nutrients for the growth of tumor cells through the formation of new blood vessels (Jiang et al., 2020). In exosomes derived from GC, the significantly elevated expression of the X26 nt target VE-cadherin promoted angiogenesis and vascular permeability (Chen et al., 2021). Exosomes produced by GC cells involved in peritoneal metastasis exhibit a decreased level of miRNA-486-5p, promoting the EMT process and facilitating peritoneal metastasis (Lin et al., 2021). Additionally, researchers have shown that miRNA-196a-1 contained in exosomes from GC cells can mediate the transfer of invasive cells between highly invasive and minimally invasive cells (Feng et al., 2019). Studies have shown that ascites-derived exosomes enhanced tumor invasiveness and neo-angiogenesis. A common conclusion from these studies is that exosomes serve as carriers for malignant behaviors in tumors (Hyung et al., 2023).

Chemotherapy is the primary approach for the late-stage treatment of GC patients (Johnston and Beckman, 2019). Drug resistance is one of the reasons for poor patient prognosis, and exosomes can serve as tools for the treatment of drug resistance. Studies have shown that GC cells resistant to cisplatin can transfer the resistant phenotype to recipient cells through exosomes. The mechanism involves miR-769-5p in exosomes regulating Caspase9, promoting the ubiquitination and degradation of p53, thus achieving drug resistance (Jing et al., 2022). Resistant GC cells can transfer drug resistance through miRNA-501 in the exosomes they produce (Liu X. et al., 2019).

Given the significant role of GC cell-derived exosomes in the progression of GC, researchers have wondered whether controlling the secretion of exosomes could be a way to control the progression of GC. It has been observed that reducing the expression level of Rab27b can decrease exosome secretion, thereby alleviating peritoneal metastasis in GC. Additionally, exosomes from other organs have been found to induce cancer-derived exosomes apoptosis (Chen et al., 2023b). This once again confirmed that exosomes from different sources have distinct biological functions.

Exosomes are also intermediate mediators of other molecules that regulate the development of cancer. Study reported that when RAB31 expression was decreased, there was a decrease in both the quantity and size of exosomes released by GC cells, as shown by exosome nanoparticle tracking analysis and electron microscopy. This discovery demonstrated that RAB31 regulates exosome secretion, a critical function in GC metastasis (Wu et al., 2023).

Exosomes may also serve as tools that are actively expelled by cancer cells and are unfavorable for cancer progression. Researchers have shown differential expression of miRNA-410-3p in exosomes from the supernatant of primary GC tissue and cultured GC cells. Increased expression levels of miRNA-410-3p were shown to inhibit the progression of primary GC (Liu and Chu, 2023). However, this raises the question of whether tumor-suppressive miRNAs can be targeted for drug delivery. This still needs to be approached with caution, as in the case of this study, while miRNA-410-3p acts as an anticancer gene in GC, it may function as an oncogene in other organs, such as the liver or lungs. Different miRNAs may play anticancer roles in one type of cancer due to distinct biological principles, while in another type of cancer, the same pathway could be procarcinogenic (Alessandrini et al., 2018; Xu et al., 2023).

### 3.3 GC exosomes and the composition and function of the GC TME

**FIGURE 3:**
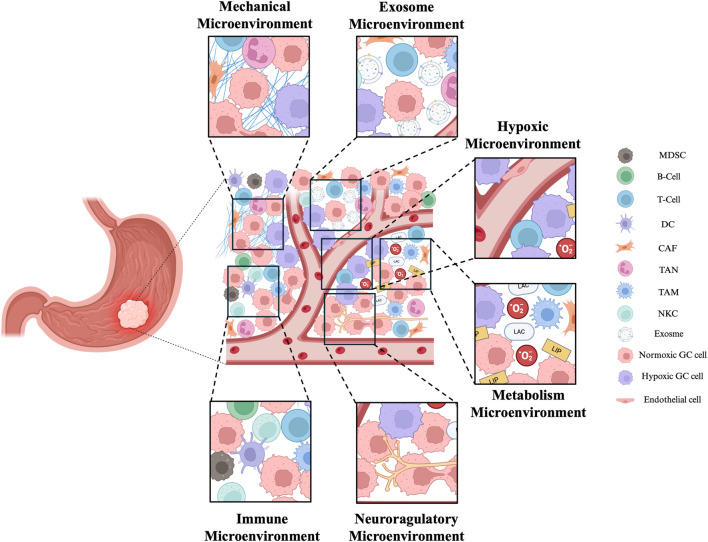
Exosomes have important roles in the TME. The complex TME in GC includes mechanical microenvironment, exosome microenvironment, hypoxic microenvironment, immune microenvironment, metabolism microenvironment and neuroregulatory microenvironment. Together, they affect the development of tumors. MDSC (Myeloid-derived suppressor cell), DC (Dendritic cell), CAF (Cancer-associated fibroblast), TAN (Tumor-associated neutrophile),TAM (tumor-associated macrophage), NK (natural killer cell). The figure was created by Biorender (https://www.biorender.com/).

The concept of the TME originated from the “seed and soil” theory and the relationship between inflammation and cancer (Paget, 1989). The TME constitutes the stroma of the tumor and consists of the tumor lesion itself, surrounding malignant or nonmalignant cells, blood vessels, immune cells, nerves, cellular metabolites, and more. Its main characteristics include low oxygen levels, low nutrient levels, and high lactate levels. The TME can be subdivided into various categories, such as the hypoxic microenvironment, immune microenvironment, metabolic microenvironment, acidic microenvironment, and neural regulatory microenvironment and mechanical microenvironment (Jin and Jin, 2020). At the same time, we must not ignore the exosomal microenvironment composed of exosomes regulated by the interaction between tumor cells and surrounding cells (Figure 3).

**FIGURE 4:**
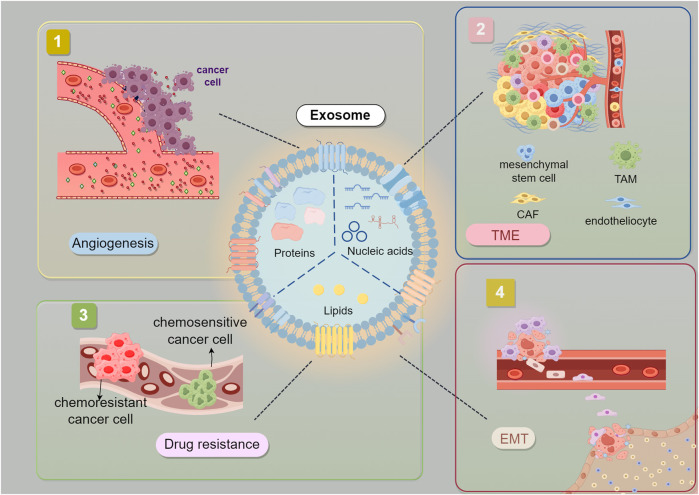
Interaction between Gastric Cancer-Derived Exosomes and TME. Exosomes derived from GC cells are believed to promote tumor EMT, angiogenesis, drug resistance. EMT (epithelial-mesenchymal transition) CAF (Cancer-associated fibroblast) TAM (tumor-associated macrophage).The figure was created by Figdraw (www.figdraw.com).

Starting from the carcinogenic mutations produced by malignant cells, surrounding non-transformed cells are recruited, releasing cytokines. Together with tumor cells, these cells mutually regulate and collectively influence tumor progression (Figure 4, Table 1) (Balkwill et al., 2012; Deng et al., 2019). The TME of GC is composed of various cell types, including the extracellular matrix (ECM), fibroblasts, endothelial cells, mesenchymal stem cells, macrophages, T cells, dendritic cells, NK, neutrophils, and endothelial cells (Pitt et al., 2016; Chhabra and Weeraratna, 2023). These cells, in addition to the extracellular components they produce, constitute the TME, and the components of the TME in GC play their respective roles in inducing immune tolerance and promoting the development of GC (Liu et al., 2022; Mao et al., 2017). A pro-cancer local inflammatory environment is formed between GC cells and fibroblasts through INHBB/NF-κB/IL-1β (Jin et al., 2023). In GC peritoneal metastasis, SOX9 in tumor cells can inhibit T-cell cytotoxicity by promoting the secretion of interleukin 6 family cytokines and promoting the polarization of M2 macrophages, creating a microenvironment conducive to tumor growth and facilitating GC peritoneal metastasis (Fan et al., 2023). Tumor-associated macrophages (TAMs) are the predominant infiltrating immune cells in the TME (Mantovani and Allavena, 2015; Noy and Pollard, 2014). Research has shown that TAMs have protumor activity and are involved in mediating immunosuppression, angiogenesis and promoting tumor resistance and metastasis in immune reactions.

**TABLE 1:** Study of exosomes in the tumor microenvironment of patients with GC.

TME factors	Exosome contents	Trends	Authors	Published year	Roles	References
Mesenchymal stem cells	miR-221	Upregulation	Wang.et al.	2014	Promoting GC cell proliferation	Wang et al. (2014)
	miR-221	Upregulation	Ma.et al.	2017	Promoting GC cell proliferation	Ma et al. (2017)
	miR-374a-5p	Upregulation	Ji.et al.	2023	Promoting GC metastasis	Ji et al. (2023)
Macrophages	miR-223	Upregulation	Zheng.et al.	2020	Promoting gastric cancer metastasis	Zheng et al. (2020a)
	miR-487a	Upregulation	Yang.et al.	2021	Promoting GC cell proliferation	Yang et al. (2021b)
	miR-513b-5p	Upregulation	Zhang.et al.	2024	Promoting GC metastasis	Zhang et al. (2024b)
Fibroblasts	MMP11	Upregulation	Xu.et al.	2019	Promoting GC metastasis	Xu et al. (2019)
	miR-139	Downregulation	Xu.et al.	2019	Promoting GC metastasis	Xu et al. (2019)
Peritumoral tissue	Has_circ_0000437	Upregulation	Shen.et al.	2022	Promote the occurrence and metastasis of GC	Shen et al. (2022a)
GC cells under hypoxic conditions	PCGEM1	Upregulation	Piao.et al.	2021	Promoting GC metastasis	Piao et al. (2021)
	miRNA-301a-3p	Upregulation	Xia.et al.	2020	Promoting GC metastasis	Xia et al. (2020)
	miRNA-199a-3p	Upregulation	Li.et al.	2023	Promoting GC metastasis	Li et al. (2023a)

Abbreviations: MMP11, Matrix Metallopeptidase 11; PCGEM1, Prostate cancer gene expression marker 1.

Research has revealed that cells present in the TME can regulate GC cells through exosomes. For instance, exosomes secreted by GC mesenchymal stem cells, which contain miR-221 and miR-374a-5p, promote the proliferation of GC cells (Wang et al., 2014; Ma et al., 2017; Ji et al., 2023). The research also demonstrates that ferroptosis-mediated oxaliplatin resistance and malignant transformation in GC are encouraged by the loss of cancer-associated fibroblast-derived exosomal DACT3-AS1 (Qu et al., 2023). Increased platelet levels also enhance the ability of bone marrow-derived mesenchymal stem cells (BMSCs) to promote cancer metastasis (Wang Q. et al., 2018). E Exosomes highly enriched with miRNA-223 from macrophage-derived exosomes can boost the migration and invasion abilities of GC cells (Zheng P. M. et al., 2020). Activated fibroblasts in the TME release exosomes overexpressing MMP11, and knocking down MMP11 has been confirmed to be related to the migration of GC cells (Xu et al., 2019). Interestingly, exosomes secreted by activated fibroblasts contain the miRNA-139, which is negatively correlated with MMP11 and can inhibit tumor progression by suppressing MMP11. GC cells secrete exosomes containing PKM2, activating the NF-κB pathway in fibroblasts and inducing an immunosuppressive microenvironment in GC (Fan et al., 2019). In addition to specific cell types, exosomes enriched with the circRNA hsa_circ_0000437 from adjacent tissues have been found to regulate the proliferative capacity of GC cells (Salunkhe et al., 2020). Melatonin has been shown to inhibit the invasion and proliferation of GC cells by modulating the expression of miRNA-27b-3p in GC exosomes (Zhang Y. Q. et al., 2023).

Hypoxia is a common microenvironment in the TME, and recent research has suggested that hypoxia in the TME may also be an indirect factor regulating the function of tumor exosomes. For instance, tumor-derived exosomes generated under hypoxic conditions have been found to promote tumor metastasis by mediating M2 macrophage polarization, and this effect is regulated by exosomal miRNA-301a, which modulates the PTEN/PI3Kγ pathway (Wang et al., 2018b). Researchers simulated a hypoxic environment under 1% oxygen and found that exosomes derived from GC cells cultured under hypoxic conditions enhanced the invasive capacity of GC cells cultured under normoxic conditions (20% O_2_). Subsequently, this phenomenon was found to be mediated by the overexpression of the lncRNA prostate cancer gene expression marker 1 (PCGEM1) in exosomes (Piao et al., 2021). Exosomes released from GC-derived exosomes under hypoxic conditions containing miRNA-301a-3p can promote the malignant behavior of GC by inhibiting HIF-1α degradation (Xia et al., 2020). Similarly, miRNA-199a-3p, which is released from exosomes under hypoxic conditions, promotes GC metastasis through MAP3K4 (Li L. et al., 2023).

Exosomes produced by GC cells can also regulate cells in the TME. For instance, researchers have shown that exosomes derived from GC cells accumulate extensively in the lungs and are phagocytosed by macrophages. This process induces immunosuppression in macrophages and increases PD-L1 expression, establishing a Pre-metastatic niche (PMN) for GC lung metastasis (Wang Y. et al., 2021). miR-605-3p suppresses vesicle-associated membrane protein 3 (VAMP3) expression, decreases exosome secretion, and concurrent inhibition of nitric oxide synthase 3 (NOS3) synthesis in cells, resulting in reduced NOS3 levels in exosomes. This process impeding multivesicular body trafficking to the cell membrane, thereby inhibiting tumor angiogenesis and PMN, consequently delaying the advancement of liver metastasis in GC (Hu et al., 2024). This regulatory effect is reciprocal, as exosomes from M2 macrophages containing miRNA-487a can promote GC progression (Xin et al., 2023). Exosomes from TAMs that carry miRNA-513b-5p promote GC progression (Zhang R. et al., 2024). GC exosomes also influence the gene expression of CD8^+^ T cells, shaping an appropriate TME for lung metastasis (Liu et al., 2020).

Exosomes secreted by GC cells can shape cells in the TME. One study indicated that GC cells from lymph node metastases regulate stromal stem cells at metastatic sites through exosome secretion to promote the malignant progression of GC (Wang M. et al., 2021). Another study revealed that GC cells can induce M2 polarization through exosomes, which results in the formation of a microenvironment conducive to tumor progression (Xin et al., 2021). GC cells induce glucose metabolism reprogramming of neutrophils through exosomes via the HMGB1/NF-κB pathway. This process mediates neutrophil N2 polarization by inhibiting SLIT2 expression through miR-4745-5p/3911, ultimately promoting GC metastasis (Zhang J. et al., 2024). Research has revealed that M2 TAMs‐derived exosomes transported MALAT1 to GC cells, where MALAT1 interacted with β‐catenin and inhibited its ubiquitination and degradation through β‐TRCP. Furthermore, MALAT1 upregulated HIF-1α expression by acting as a sponge for miR-217-5p. M2-type TAM exosomes activate β-catenin and HIF-1α signaling pathways together to enhance aerobic glycolysis of GC cells (Wang et al., 2024).

In this section, we summarize recent research on the biological functions of GC exosomes. We also highlighted the role of exosomes from the TME in GC, including those produced by different cell types within the microenvironment. The composition of the TME is complex, with hypoxia and immune components being the two main signatures of the TME. These findings may lead to potential targets for exosome-based therapeutic strategies. Understanding the interplay between exosomes and various TME subtypes could enhance the combined therapeutic potential of exosomes. Research has suggested that exosomes in the TME are diverse, as almost every cell type in the TME can secrete exosomes (Yang et al., 2020). The potential interactions between the regulatory effects of these exosomes are a topic that warrants further investigation.

## 4 GC exosomes in clinical applications

### 4.1 As early diagnosis biomarkers

A low diagnostic rate, high metastasis rate, and high recurrence rate are common factors contributing to the poor prognosis of GC patients (Thrift and El-Serag, 2020). Currently used tumor markers for the clinical diagnosis of GC lack sensitivity and specificity. There are few clinically validated markers related to metastasis. As a potential noninvasive diagnostic tool, exosome biopsy has shown significant value in the early diagnosis and prognostic assessment of GC (Table 2). New and more sensitive screening strategies have also been proposed (Lopez et al., 2018).

**TABLE 2:** Potential diagnostic molecular markers in GC exosomes.

Type	Name	Source	Authors	Publication year	Roles	References
miRNA	miR-134	Serum	Jin.et al.	2022	GC diagnosis and prognostic prediction	Jin et al. (2022)
	miR-92a-3p, miR-379-5p	Serum	Lu.et al.	2021	GC diagnostic markers	Lu et al. (2021)
	miR-410-3p, miR-19b-3p	Serum	Liu.et al.	2022	Prediction of GC metastasis	Liu and Chu (2020)
	miR-16p-5p	Plasma	Zhang.et al.	2015	Assessment of GC Progression	Zhang et al. (2015)
	miR-769-5p	Culture medium	Jing.et al.	2022	Assessment of GC drug resistance	Jing et al. (2022)
	miR-29s	Peritoneal fluid	Ohzawa.et al.	2020	Prediction of Tumor Recurrence	Ohzawa et al. (2020b)
lncRNA	HOTAIR	Serum	Chen.et al.	2023	Predicting GC metastasis	Chen et al. (2023a)
	lnc-GNAQ-6:1	Serum	Li.et al.	2020	GC diagnostic biomarkers	Li et al. (2020b)
	lnc-SLC2A12-10:1	Plasma	Zheng.et al.	2020	GC diagnosis and prognostic prediction	Zheng et al. (2020b)
cRNA	Has_circ_0000437	Serum	Shen.et al.	2022	Prediction of GC metastasis	Shen et al. (2022a)
	circ-RanGAP1	Plasma	Lu.et al.	2020	Prediction of GC prognosis	Lu et al. (2020)
	circFCHO2	Serum	Zhang.et al.	2022	Diagnostic markers for GC	Zhang et al. (2022)
	has_circ_000200	Serum	Huang.et al.	2023	Assessment of GC progression	Huang et al. (2023)
	has_circ_0000419	Plasma	Tao.et al.	2019	Diagnosis and Prognostic prediction of GC	Tao et al. (2020)
	has_circ_0015286	Plasma	Zheng.et al.	2022	GC diagnosis and prognostic prediction	Zheng et al. (2022)
	CDR1as	Plasma	Li.et al.	2023	GC diagnosis and prognostic prediction	Li et al. (2023b)
	cric-KIAA1244	Plasma	Tang.et al.	2018	Prediction of GC metastasis	Tang et al. (2018)
	Circ50547	Plasma	Zang.et al.	2024	GC diagnosis and prognostic prediction	Zang et al. (2024)
protein	TGF-β1, ILK1	blood	Yen.et al.	2017	Prediction of GC Metastasis	Yen et al. (2017)
	CD14	Plasma	Zhou.et al.	2023	Prediction of GC Metastasis	Zhou et al. (2023)
	TRIM3	Serum	Fu.et al.	2018	GC diagnostic markers	Fu et al. (2018)

Abbreviations: HOTAIR HOX, antisense intergenic RNA, CDR1 as cerebellar degeneration-related protein 1 transcript, TGF-β1, Transforming growth factor-beta1, ILK1 Integrin-linked kinase 1, TRIM3, Tripartite motif-containing 3.

In theory, the contents of exosomes have the potential to serve as detection markers. Research has focused extensively on miRNA as a predictive marker (Chen et al., 2020). However, studies have also analyzed the proteomics of exosomes to determine their tumor metastatic potential (Chen et al., 2023c). Several miRNAs, including miRNA-134 (Jin et al., 2022), miRNA-23b (Zhang R. et al., 2024), and serum miRNA-92a-3p, are suitable detection markers (Lu et al., 2021). Additionally, miRNAs within exosomes hold promise as biomarkers for assessing postoperative metastatic risk in GC patients. For instance, in a study analyzing serum samples from 89 stage II/III GC patients, researchers found that elevated levels of exosomal miRNA-379-5p and miRNA-410-3p were associated with shorter progression-free survival (Liu and Chu, 2020). Circulating exosomal miRNA-19b-3p and miRNA-106p-5p in GC patients may be related to cancer staging and lymphatic metastasis (Zhang et al., 2015). The previously mentioned miR-769-5p might also serve as a potential biomarker for assessing GC drug resistance (Jing et al., 2022).

Proteins in exosomes have also been found to potentially serve as predictive markers, although relatively fewer studies have evaluated these proteins than miRNAs. Researchers isolated exosomes from gastric serosal venous samples of 61 GC patients and found that the expression level of TGF-β1 in exosomes is associated with lymphatic metastasis in GC (Yen et al., 2017). In another study, researchers collected peripheral blood serum from late-stage GC patients with metastasis and extracted and purified exosomes for analysis and discovered that ILK1 and CD14 can influence the colony-forming ability of GC cells and are associated with organ-specific metastasis (Zhou et al., 2023). Proteins in exosomes are primarily membranes and glycoproteins. Given the characteristics of exosome formation, exosomal membrane proteins are related to the source of target cells. Analysis of the total protein profile of serum exosomes from healthy controls and GC patients revealed that TRIM3 could be a potential biomarker for GC diagnosis (Fu et al., 2018). PD-L1 in GC exosomes can reflect the immune status of GC patients (Shen D. D. et al., 2022). Based on these findings, future studies could investigate whether different membrane protein markers or miRNAs are associated with specific organ metastasis or the metastatic growth characteristics of cancer cells (Zhou et al., 2023).

LncRNAs also have the potential to serve as potential liquid biopsy markers GC growth and metastasis. For example, as mentioned earlier, HOTAIR can be detected in both serum and tumor tissue (Zhou et al., 2023). lnc-GNAQ-6:1 is considered a potential marker for exosomal liquid biopsy in GC patients (Li S. et al., 2020), and the serum exosomal lnc-SLC2A12-10:1 can also be used for diagnosis and prognosis monitoring (Zheng P. et al., 2020). Furthermore, there was a strong correlation found between the invasion depth and TNM stage and the elevated expression of serum exosomal HOTTIP in GC (Zhao et al., 2018). Comparing exosomal HOTTIP to traditional biomarkers CEA, CA19–9, and CA72-4, the former showed superior diagnostic accuracy. Also, a negative overall survival rate was highly linked with elevated exosomal HOTTIP levels, establishing exosomal HOTTIP as a separate prognostic factor in GC patients. Research on circRNAs in GC -related tumors started relatively late, but researchers have found that they also have the potential to be detection markers. For instance, the previously mentioned circRNA Has_circ_0000437 can be detected in patient serum (Shen X. et al., 2022). Circ-RanGAP1 is upregulated in the serum exosomes of GC patients (Lu et al., 2020) and promotes tumor progression by regulating VEGFA. CircFCHO2 can also be detected in the serum of GC patients (Zhang et al., 2022). Like the aforementioned circRNAs, those verified in studies include hsa_circ_000200 (Huang et al., 2023), hsa_circ_0000419 (Tao et al., 2020), and hsa_circ_0015286 (Zheng et al., 2022). Some circRNAs can also serve as indicators for assessing the prognosis and metastatic risk of GC patients. For example, the circRNA CDR1as may be a prognostic marker for GC (Li R. et al., 2023), and researchers have found that the circRNA KIAA1244 can predict the risk of GC lymph node metastasis (Tang et al., 2018).

### 4.2 As prognostic indicators

Tumor tissue pathology is currently the gold standard for diagnosing GC and enables the analysis of patient tumor stage and the selection of appropriate treatment strategies based on pathological results. Liquid biopsy is not intended to replace pathological examination but serves more as a supplementary choice for investigation. For instance, certain small-molecule markers have been found to have the potential to predict the preferred metastatic site and prognosis of tumors. Therefore, exosomal liquid biopsy as a complementary auxiliary examination is meaningful. Notably, the heterogeneity of exosomes derived from blood may affect the accuracy of detection (Lopez et al., 2018). Changes in a patient’s general condition can influence exosome secretion and content alterations. Therefore, a meticulous experimental design is required to address these considerations.

Serum exosomal liquid biopsy is not necessarily the sole approach for exosomal analysis. Peritoneal lavage fluid or ascites can also serve as alternative sources for sample collection (Ohzawa et al., 2020a; Tokuhisa et al., 2015; Hu et al., 2019). The miRNA-29s in peritoneal fluid are predictive factors for tumor recurrence (Ohzawa et al., 2020b). Exosomal biomarkers such as has_let_7g_3p and has_miR_10395-3p in peritoneal lavage fluid can predict the occurrence of peritoneal metastasis and systemic chemotherapy efficacy (Luo et al., 2023). Exosomal miRNAs from neutrophils act as accurate biomarkers for GC diagnosis (Yu et al., 2024)**.** Moreover, researchers have isolated exosomes from gastric juice, expanding the possibilities for sample sources (Kagota et al., 2019). Currently, in liquid biopsy studies related to tumor exosomes, sample sources include saliva, serum, urine, etc. However, whether exosomes from different sources have sufficient representativeness remains a question for further investigation when considering practical clinical applications. It is crucial to determine which sample type provides better representativeness and stability in detection through additional research.

### 4.3 As therapeutic target

Exosomes, serving as innovative vehicles for cancer vaccines, present a more promising approach than conventional vaccines because of their reduced immunogenicity, tumor heterogeneity accommodation, and superior delivery efficiency. These vesicles exhibit inherent low immunogenicity, precise targeting abilities, biological durability, and intercellular communication attributes, rendering them well-suited for cancer vaccine advancement. They have the capacity to transport bioactive substances like DNA, ncRNAs, and proteins that modulate the immune characteristics of recipient cells. Furthermore, exosomes can convey tumor neoantigens and immune stimulants, triggering persistent and wide-ranging anti-tumor immune reactions (Sheikhhossein et al., 2024).

In cancer immunotherapy, tumor-derived exosomes (TEX) as potent cell-free peptide-based antitumour vaccine through dendritic cell-released MHC class I/peptide complexes for efficient CD8^+^ T cell priming to suppress tumour growth or prime naive Tc1 lymphocytes leading to tumor rejection (Andre et al., 2004; Chaput et al., 2004). With additional preclinical and clinical validation, the TEX vaccine may emerge as a potential tool in the fight against cancer. Notably, Zitvogel et al. pioneered the investigation of tumor suppression through exosome-based vaccines in 1998 (Zitvogel et al., 1998). The major research applications of TEX Vaccinnes in solid tumors including lung cancer (Yaddanapudi et al., 2012) and breast caner (Xie et al., 2018). However, the application of these treatments in GC is slow to progress.

### 4.4 Drug delivery system

Recently, exosomes have garnered significant interest in serving as natural nanocarriers for delivering anti-GC drugs due to their high biocompatibility, low immunogenicity, ability to target tumor cells, enhance drug efficacy, and mitigate adverse reactions. Researchers have demonstrated the important role of SALL4 in enhancing tumor angiogenesis through the modulation of VEGF expression. It also suggested an exosome-based nano-drug delivery system loaded for the concurrent administration of siRNA directed against the SALL4/VEGF pathway and the anticancer medication thalidomide. The study validated the prospective utility of the exosomal drug delivery system in anti-angiogenic and therapy of GC by suppressing SALL4/VEGF pathway (Abouelnazar et al., 2023). Research on a novel delivery strategy was found to transfect synthetic miR-200a mimics into exosomes via electroporation. In a mechanistic way, miR-200a increased E-cadherin water and decreased the expression levels of β-catenin, vimentin, ZEB1 and Snail1, leading to EMT inhibition in GC cells (Mirzaei et al., 2023). In addition, researchers proposed that 17-DMAG-loaded MKN45-targeting exosomes effectively deliver 17-DMAG to GC cells, resulting in enhanced antitumor effects. This research aims to provide insights into the design of targeted exosome-based therapies for GC in terms of potential clinical applications (Park et al., 2024).

### 4.5 Chemoresistance

Recent studies have identified exosomes as mediators of tumor chemotherapy resistance through various mechanisms. These mechanisms include direct drug efflux, transportation via drug efflux pumps, and intercellular exchange of miRNA. Moreover, exosomes contribute to tumor chemotherapy resistance by facilitating the activity of efflux pump transporters such as P-glycoprotein(P-gp), multidrug resistance protein-1 (MRP-1), ATP-binding cassette transporter A3 (ABCA-3), and ATP-binding cassette transporter G2 (ABCG-2) (Shedden et al., 2003; Levchenko et al., 2005; Ambudkar et al., 2005). Exosomes loaded with miRNAs, mRNAs, and other ncRNAs can regulate drug resistance. Researchers explored the potential therapeutic approach of exosome delivery of anti-miR-214 to reverse cisplatin (DDP) resistance in GC. They confirmed that anti-miR-214 could enhance the sensitivity of GC cells to DDP by cell experiments. Then they constructed and verified that exosome-loaded anti-miR-214 (Exo-anti-214) could effectively enhance the sensitivity of GC cells to DDP by down-regulating the expression of tumor miR-214 and up-regulating the related target proteins. This study provides a new therapeutic strategy based on a combination of systemic injection of exosomes containing anti-miR-214 and intraperitoneal DDP administration was proposed for overcoming DDP-resistant GC (Wang et al., 2018c). In GC resistant cell line MGC-803/5-FU, the expression level of TFAP2E was decreased due to hypermethylation, which directly promoted the chemotherapy resistance to 5-FU. Treatment with the demethylating drug 5-aza-2 ′-deoxycytidine (5AZA) can reverse this effect. miR-106a-5p and miR-421 are significantly overexpressed in exosomes secreted by drug-resistant cells, which are involved in mediating the methylation-related resistance mechanism of TFAP2E by targeting regulatory genes such as E2F1, MTOR, and STAT3. After co-culture with sensitive cells, exosomes may enhance drug resistance by delivering these miRNAs (Jingyue et al., 2019). Similarly, miR-500a-3p was significantly highly expressed in DDP-resistant GC cells and their secreted exosomes. Drug-resistant cells deliver miR-500a-3p to sensitive cells via exosomes, inhibiting the expression of tumor suppressor FBXW7 and enhancing the DDP resistance and stemness of recipient cells. These study revealed the key role of exosomes and their miRNAs in chemotherapy resistance (Lin et al., 2020). A recent study revealed a new mechanism by which M2-polarized macrophages deliver miR-3681-3p via exosomes to promote DDP resistance in GC cells. Experiments have shown that M2 macrophage-derived exosomes target miR-3681-3p to inhibit the expression of MLH1, a key of DNA repair protein, and significantly enhance DDP resistance in GC cells, while MLH1 overexpression can reverse drug resistance (Wei et al., 2025). Similarly, research demonstrated that the exosome secreted by M2 macrophages delivered circTEX2 to GC cells and relieved its inhibition of drug effector protein ABCC1 by adsorption of miR-145, thus enhancing the resistance of tumor cells to DDP. These study suggested that targeting exosome-dependent signaling pathways between M2 macrophage and cancer cells may be a potential therapeutic strategy to overcome DDP resistance in GC (Qu et al., 2024).

## 5 Discussion

**FIGURE 5:**
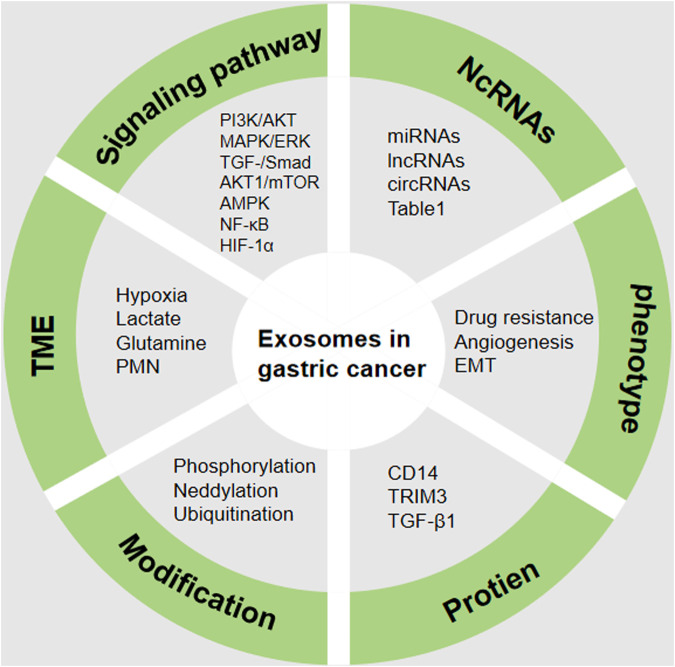
Regulatory mechanism of exosomes in GC. The complex mechanism in GC includes the aberrant expression of NcRNAs, protein, phenotype, signaling pathway, TME and modification. TME (tumor microenvironment), NcRNAs (Non-coding RNAs), EMT (Epithelial-mesenchymal transition), PMN (Pre-metastatic niche), HIF-1α (hypoxia inducible factor-1), AMPK (Adenosine 5′-monophosphate -activated protein kinase), NF-κB (nuclear factor-kappa B). The figure was created by power point.

Cancer is a complex systemic disease involving the participation of the immune system, vascular system, nervous system, and other factors in its onset and progression. As the primary mode of intercellular communication, exosomes possess the capacity to influence the fate of both their originating cells and recipient cells. Their impact on tumors manifests in various ways, including stimulation of tumor growth and resistance to drugs, initiation of myofibroblast differentiation, facilitation of angiogenesis, promotion of PMN formation, and induction of immunosuppression. Extensive research has demonstrated the involvement of exosomes in critical biological processes such as angiogenesis, proliferation, invasion, migration, and recurrence in GC. Moreover, exosomes play a pivotal role in modulating the drug resistance of GC cells, thereby advancing the disease. The presence of diverse bioactive molecules within tumor cell-derived exosomes positions them as promising candidates for early GC detection (Figure 5). Furthermore, the potential of exosomes to serve as targeted drug delivery vehicles underscores their significant future applications. In previous overall landscape of research on exosome regulation of tumor progression and growth, there is a predominant focus on miRNA studies. This is evident not only in the biological functions of miRNAs but also in the noninvasive early diagnosis of tumors through liquid biopsy using characteristic miRNA profiles. With technological advancements, there is a growing recognition of the ability of exosomes to transport various molecules for intercellular communication with a range of cell types, including mesenchymal stem cells and different immune cells. Simultaneously, some scholars have raised questions about the physiological functions of exosomes. For instance, concerns have been raised about whether the quantity of exosomes is sufficient to generate biological functions. Consequently, manipulating the exosome biogenesis process to enhance the production of exosomes/EVs with specific functions in targeted cell types holds significant promise for clinical utility. Additionally, there are questions about whether exosomes, when used as exogenous drugs, can evade recognition by immune cells. Future researchers should delve deeper, considering a systemic organ perspective and comprehensively assessing the role of tumor exosomes. Furthermore, due to the specific location of primary GC occurrence, certain microbiota in the gastrointestinal tract may also produce exosomes, influencing the onset and progression of GC. For instance, *H. pylori* has been found to inhibit the immune response gamma-glutamyltransferase (GGT) through exosome secretion (Franzini et al., 2014). These findings suggest that when exosome-related studies are conducted in GC patients with concurrent bacterial infections, the situation may become more complex, and the mixture of various factors could lead to inaccurate results.

## 6 Conclusion

Exosomes play a pivotal role in GC progression, acting as key regulators in tumor growth, metastasis, and immune evasion. They carry a variety of bioactive molecules, including miRNAs, lncRNAs, circRNAs, and proteins, which can serve as potential biomarkers for early diagnosis and prognosis in GC. The interaction between GC-derived exosomes and the TME is complex, with exosomes from various cellular components influencing tumor behavior. Engineered exosomes offer a promising therapeutic strategy by delivering anticancer drugs directly to tumor sites. Further research is needed to fully understand the multifaceted roles of exosomes in GC and to develop effective exosome-based diagnostic and therapeutic approaches.

## 7 Future directions

In the context of exosome therapeutic strategies, engineering exosomes may be a promising approach (Zhang M. et al., 2023). Engineering modifications of exosomes can enable them to carry specific drugs, enhance their targeting to cancer cells, and evade immune surveillance. These advantages make engineered exosomes therapy potentially more advantageous than traditional methods. Scientists have employed various contemporary techniques such as transmission electron microscopy, dynamic light scattering, nanoparticle tracking analysis, protein quantification, Western blotting, and flow cytometry to characterize exosomes. However, due to the alterations in exosomes morphology, purity, and biological function during isolation and processing, their characterization remains a challenging and pivotal issue in current research. Consequently, there is a huge amount of space for further exploration and advancement in understanding the composition and attributes of exosomes. Enhanced methodologies are essential for the comprehensive characterization and analysis of exosomes. Exosomes serve as a double-edged sword for tumor cells. Exosomes from tumor cells typically promote cancer, while those derived from immune cells often have anticancer functions. Distinguishing and defining exosomes secreted by different subtypes of cells are critical aspects of future research. Currently, exosomes extraction and identification are primarily performed in cells, limiting research within the organism. Encouragingly, researchers are exploring techniques to identify exosomes in tissues (Crescitelli et al., 2021). It is anticipated that future studies can isolate exosomes from tissues, bringing related research closer to clinical applications. Despite the broad potential of exosomes as drug delivery vehicles, challenges remain regarding their yield, stability, targeted delivery efficiency, and immunogenicity. Exosomes inherently transport biologically active molecules from their parent cells. However, using them directly for therapeutic purposes may induce off-target effects or increase the risk of metastasis. Therefore, experts advocate for the implementation of rigorous standards for exosome modification and quality control to improve targeting through surface modifications (e.g., targeted peptide coupling) or content editing (e.g., CRISPR-mediated regulation of specific RNA) and to mitigate off-target risks. With the emergence of AI/ML, it is believed that future exosomes can better serve the clinic.
